# Echocardiography in the Assessment of Valve Regurgitation– Incremental Role of Three Dimensional Echocardiography

**DOI:** 10.31083/j.rcm2312407

**Published:** 2022-12-14

**Authors:** Hong Lee, Tasneem Z Naqvi

**Affiliations:** ^1^Department of Medicine, Banner University Medical Center, Phoenix, AZ 85719, USA; ^2^Department of Cardiovascular Disease, Division of Echocardiography, Mayo Clinic, Scottsdale, AZ 85259, USA

**Keywords:** valve regurgitation, echocardiography, etiology, valve repair, Doppler echocardiography, three-dimensional echocardiography

## Abstract

Echocardiography (Echo) has a primary role in the evaluation of cardiac valve regurgitation. Echo valve regurgitation assessment includes multiple qualitative and quantitative methods which require adequate image quality, comprehensive echocardiographic images and precise measurements to obtain accurate assessment. For patient management, it is also important to investigate the mechanism of valve regurgitation. Severity and mechanism of valve regurgitation determine whether continued medical follow up is optimal or surgical or percutaneous valve repair, or replacement option should be necessary. Transthoracic Echo (TTE) is the gold standard most commonly used for the assessment of valve leaflet anatomy, valve motion and regurgitation severity to determine primary versus secondary causes of valve regurgitation, however transesophageal echo (TEE) provides high resolution imaging of valve leaflets and supporting apparatus and oftentimes determines mechanism of valve regurgitation particularly for mitral and tricuspid valves when TTE is unable to determine the mechanism. By providing surgical type views in a moving heart under normal hemodynamic conditions, 3D TEE has greatly improved assessment of mechanism and etiology of valve regurgitation. Besides, TEE also allows quantitation of valve regurgitation severity by Doppler methods as well as direct 3D planimetry of valve area and regurgitant orifice area. Doppler methods are pre and afterload dependent whereas direct 3D planimetry allows assessment of location and severity of valve regurgitation irrespective of ventricular loading conditions. Pre or intraoperative 3D TEE assessment can provide valuable information for surgical planning of valve repair or replacement. This review discusses various valvular pathologies causing regurgitation and the role of TTE and TEE in improving this assessment as shown by several case examples.

## 1. Introduction

Echocardiography (Echo) plays a primary role in the evaluation of cardiac valve regurgitation severity. This assessment includes multiple qualitative and quantitative methods for which comprehensive echocardiographic images of good quality, and precise echo measurements are essential for most accurate assessment. For patient management, is very important to understand the mechanism of valve regurgitation to determine whether surgical or percutaneous methods for treatment of valve regurgitation should be considered or medical treatment is the appropriate method of treatment. Transthoracic echo (TTE) allows assessment of valve leaflet anatomy, valve motion and regurgitation severity, however transesophageal echocardiography (TEE) provides high resolution imaging and 3D TEE provides surgical type views thereby improving assessment of etiology of valve regurgitation such as leaflet restriction, flail, perforation, calcification, infection, or impingement from pacemaker/intracardiac defibrillator (ICD) leads for tricuspid valve or normal leaflet anatomy but primary cardiac myopathic process or dilation of supporting apparatus. This review discusses assessment of severity and etiology of valve regurgitation by TTE and TEE and the role of 3D TEE in further improving this assessment. Several case examples are provided to demonstrate the utility of 2D and 3D TTE and TEE.

## 2. Mitral Valve 

Mitral valve apparatus comprises of mitral valve leaflet, mitral annulus, chordae tendinea and papillary muscles which are attached to the left ventricular myocardial walls. Mitral valve leaflets comprise of a wider anterior and a narrower posterior leaflet which however occupies greater circumference of the mitral annulus. The posterior leaflet has 3 distinct scallops, P1, P2 and P3 from anterior to posterior. The anterior leaflet is usually a large single scallop, however, for practical purposes it is divided into three scallops (A1, A2, A3). The anterior and posterior mitral annulus where the leaflets meet forms the anterior and posterior commissure respectively. The anterior and posterior leaflet coaptation plane is closer to the posterior than anterior leaflet due to larger width of the anterior leaflet.

Based on Carpentier Classification, the etiology, mechanism, cardiac remodeling and recommended treatment approaches for mitral regurgitation (MR) are described in Table 1.

**Table 1.** **Classification of etiology and mechanism of mitral valve regurgitation by Carpentier Classification**.

Carpentier Classification		Type of MR	Ventricle	Leaflet motion	Pathology	MR jet	Management
Type I	Acquired leaflet damage	Primary	Normal	Normal	Perforation, vegetation	Single or multiple jets through the leaflet/s	Valve replacement, occ valve repair
	Dilated mitral annulus with normal LV function	Secondary	Normal	Normal	Dilated annulus in severe left atrial enlargement (atrial functional MR)	Central coaptation single or multiple jets	Mitral Annuloplasty
Type II	Degenerative leaflets Fibroelastic deficiency (FED), Barlow’s mitral valve	Primary	Normal	Excessive	Mitral valve prolapse, flail with torn chordae in FED or Barlow’s valve, papillary muscle rupture, trauma	Eccentric single jet, multiple jets may occur including central jet with involvement of multiple scallops	Surgical, Repair
Type IIIa	Acquired leaflet damage	Primary	Normal	Restriction in systole and diastole	Rheumatic, diet drugs	Central	Valve replacement usual, repair performed in some centers
Type IIIb	Dilated mitral annulus, Dilated left ventricle	Secondary	Focal or generalized LV dilation, chordal displacement and mitral leaflet tenting	Restriction in systole only	Papillary muscle displacement from ventricular remodeling in focal infarct or ischemic cardiomyopathy with chordal tethering and leaflet tenting, Functional in non-ischemic dilated cardiomyopathy	Central single or multiple coaptation jets	GDMT, CRT, percutaneous valve repair, surgical if concomitant CABG or other valve surgery

GDMT, Guideline directed medical therapy; CRT, cardiac resynchronization treatment; CABG, coronary artery bypass surgery; MR, mitral regurgitation; LV, left ventricle.

Degenerative mitral valve disease is the most common cause of MR in the United States [1]. Mitral valve repair leads to an improved patient outcome compared to mitral valve replacement and is feasible in the majority of patients.

### 2.1 Echocardiographic Assessment of Mitral Valve Structure and Regurgitation Severity

MR severity is determined by a combination of several echo parameters well delineated in the ACC/AHA/ASE and European guideline documents [2, 3]. These include anatomy of mitral leaflets and supporting apparatus, size, geometry and function of left ventricle and left atrium, size and function of right ventricle and pulmonary artery pressure. Severity of MR is assessed by qualitative, semiquantitative as well as quantitative methods. These include eyeball assessment of MR jet area within the left atrium estimating percent of left atrium occupied by the MR jet, contour and direction of MR jet, vena contracta width of MR jet, continuous-wave (CW) Doppler intensity, the shape of the transmitral regurgitant jet velocity curve, effective orifice area (ERO), regurgitant volume (RV), regurgitant fraction (obtained by using the proximal isovelocity surface area (PISA) method or quantitative Doppler flow measurements) [4], Criteria for severe MR include vena contracta width of ≥7 mm, ERO of ≥0.4 ${\mathrm{cm}}^{2}$, regurgitant volume of ≥60 mL and regurgitant fraction of ≥50%. Volumetric measurements provide a better assessment as conventional color Doppler parameter may overestimate its severity on a single image [5]. Earlier ACC/AHA guidelines reduced the cut off for EROA for severe functional MR to ≤0.2 ${\mathrm{cm}}^{2}$ based on outcome studies that have shown poor prognosis in patients with ERO ≥0.2 ${\mathrm{cm}}^{2}$ [5], however the most recent ACC/AHA guidelines suggest the same EROA (≥0.4 ${\mathrm{cm}}^{2}$) for severe MR of functional and degenerative etiology [3].

**Fig. 1.**
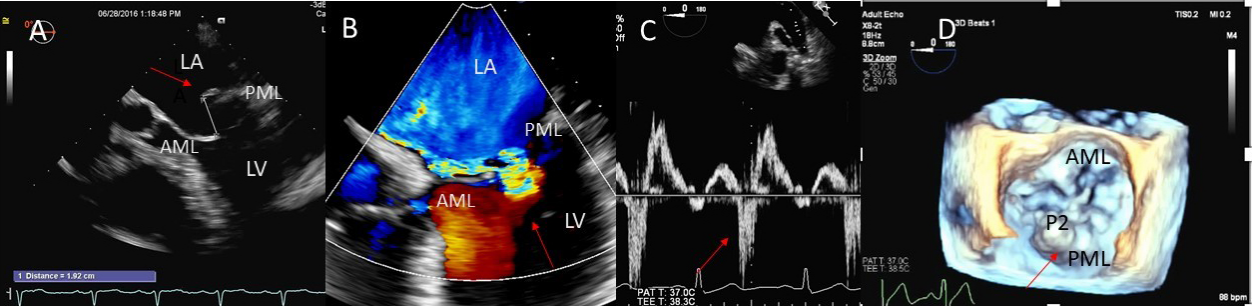
**Degenerative MR due to Flail posterior mitral valve leaflet**. (A) TEE 4 chamber view showing a flail posterior mitral valve resulting in a severe anteriorly directed mitral regurgitation jet. LA, left atrium; LV, left ventricle; AML, anterior mitral leaflet; PML, posterior mitral leaflet. (B) TEE 4 chamber view color Doppler showing anteriorly directed mitral regurgitation with PISA (red arrow). AML, anterior mitral leaflet; AV, aortic valve. (C) Pulsed wave Doppler showing left upper pulmonary vein systolic flow reversal. (D) 3D TEE enface view of the mitral valve from the atrial perspective demonstrating P2 flail scallop with torn chordae. Aortic valve is a 9 o’clock position. AML, anterior mitral leaflet; PML, posterior mitral leaflet.

These criteria are generally more reliable in central jets. Pulmonary artery systolic pressure, mitral inflow E wave velocity and pulmonary vein flow pattern are other helpful parameters. Systolic pulmonary vein reversal (Fig. 1) is highly specific for severe MR but is not very sensitive. Discrepancy may occur between MR ERO and RV in mitral valve prolapse in early stages of MR where non holosystolic MR jet duration and hence regurgitant volume are smaller than the PISA derived EROA which does not account for the duration of MR jet. 2D vena contracta width may be unreliable in eccentric jets, however direct measurement of regurgitant orifice can be done using 3D color Doppler vena contracta area which may allow better quantitation of MR in central as well as eccentric MR jets [5] as well as in patients with multiple MR jets in whom PISA quantitation by adding multiple jets has not be validated and in whom continuity equation cannot be performed [6].

### 2.2 Indications for Mitral Valve Surgery

Symptom onset is a distinctive point in developmental history of MR and also starting point for intervention. Because symptoms develop gradually, patients may fail to recognize or ignore symptoms. In patients with chronic MR, exercise echocardiography [7, 8] or exercise invasive hemodynamics may unmask exertional symptoms in asymptomatic patients. Exercise echocardiography can also be helpful when there is discrepancy between MR severity and clinical symptoms such as in patients with moderate MR. In such patients, MR and filling pressures may become significantly worse with exercise helping to demonstrate MR as the cause of the patient’s dyspnea. Among asymptomatic patients with severe MR, signs of LV remodeling and systolic dysfunction are reflected by LVESD ≥40 mm or LVEF <60% which are indications for mitral valve repair [3] provided a >90% success rate for mitral valve repair can be predicted operatively [9, 10]. The results of mitral valve repair are superior to the results of mitral valve replacement even in elderly patients [11, 12, 13] if the repair is performed at surgery centers having high surgical volume. Patients with significant MR are often not referred for surgical management [14]. Twenty-year outcome status post pre mitral repair vs. replacement for moderate to severe or severe degenerative MR showed that mitral valve repair has lower mortality than after replacement [15]. Twenty-year survival was also better after mitral valve repair than after replacement (46% vs. 23%) [15]. Mitral valve (MV) repair has been shown to be superior than replacement in patient subsets on the basis of age, sex, or any stratification criteria [15].

Elevated natriuretic peptide levels provide objective evidence of increased preload to provide an adequate cardiac output in patients with chronic severe MR and may be helpful in making treatment decisions [16]. Repair should also be considered first, with other causes of severe MR, such as papillary muscle rupture, infective endocarditis, and cleft mitral valve. Complex and extensive repair is needed for anterior and/or bileaflet primary mitral valve disease [17, 18].

Degenerative MR is the most common cause of mitral valve repair and is caused by valvular or chordal degeneration and systolic excessive leaflet movement defined by a prolapse in left atrium ≥2 mm, or flail leaflet which can affect one or both leaflets and one or multiple scallops [1] (Fig. 1A–D).

Pre or intraoperative communication between echocardiographer and the surgeon regarding MV pathology is key to successful MV repair. Surgeon’s view of the MV can be effectively reproduced with the *en face *real time view on 3D image of the MV (real time or with reconstruction). The aortic valve (AV) can be seen in the 12 o’clock position, left atrial appendage is visualized in the 9 o’clock position, and the coronary sinus is at the 3 o’clock position [19, 20]. Automation advances in 3D imaging allow rapid reproduction of this view.

**Fig. 2.**
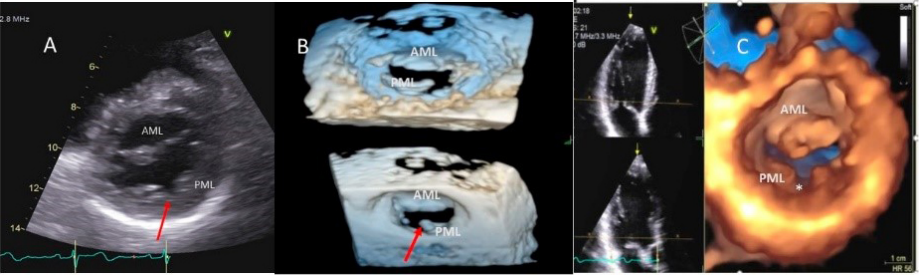
**Mitral leaflet Cleft**. (A) TTE short axis at the mitral valve level showing posterior mitral cleft. (B, C) Isolated cleft within the P2 segment of the posterior leaflet (associated with mitral regurgitation) on 3D TEE and TTE short axis views. AML, anterior mitral leaflet; PML, posterior mitral leaflet.

Besides prolapsing or flail scallops, MR jet may also originate between individual scallops. This occurs more commonly between the posterior leaflet through cleft like indentations that sometimes extend to the mitral annulus. This origin of MR can be very difficult to diagnose on 2D TTE or TEE (Fig. 2A–C). 3D color Doppler further assists in confirming jet origin at the site of suspected leaflet pathology/ies including presence of mitral valve cleft like indentation/s. Presence of calcification on the annulus and leaflets and in the subvalvular apparatus further assists surgeon in planning repair [21]. Visualization of the valve from the LV perspective adds further information on leaflet morphology, coaptation and regurgitant site/s particularly if jet originates from mitral valve clefts. Optimal visualization of the MR jets using real-time 3D TEE leads direct guidance for catheter movement and positioning of the implanted device(s) capturing the opposing sides of anterior and posterior mitral leaflet scallops during catheter based MV interventional procedures [22].

**Fig. 3.**
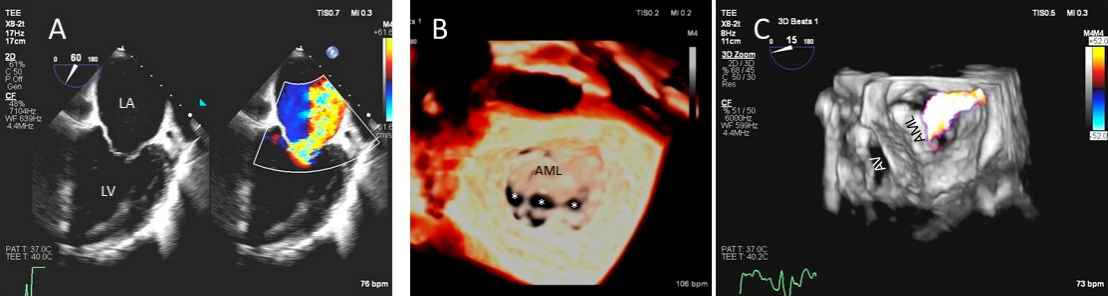
**Functional Mitral regurgitation**. (A) Transesophageal echocardiogram biplane view shows a dilated LV with mitral valve coaptation point displaced into the LV and central MR jet. (B) 3D TEE showing central mitral leaflet malcoaptation (white asterisks). (C) 3D TEE surgical view of the mitral valve showing severe central mitral regurgitation. LA, left atrium; LV, left ventricle; AML, anterior mitral.

As opposed to degenerative MR, mitral valve leaflets in secondary MR may be normal but MR results from leaflet mal-coaptation due to a dilated mitral annulus as in dilated cardiomyopathy or due to tenting of mitral leaflets due to LV infarct remodeling causing outward displacement of papillary muscles and tethering of chordae attached to these papillary muscles. Mal-coaptation may be along the entire mitral leaflet coaptation plane mostly in functional MR (Fig. 3A–C) or localized to some scallops commonly seen at the P3 scallop of the mitral valve in the presence of a remodeled infero-posterior myocardial infarction causing tethering of the chordae to P3 scallop (ischemic MR).

### 2.3 Post-Operative Follow Up

In a study evaluating surgical mitral valve repair with echo-guidance, repair was successful in 99% of degenerative, 97% of myopathic, and 84% of inflammatory mitral valve disease [23]. Concordance of echo findings with surgical findings increased the chances of successful repair by 2D TEE (98% vs. 57%) and 3D TEE (100% vs. 94%) [24]. Another study found global LV reverse remodeling after 5 years of successful MV repair [24].

## 3. Tricuspid Valve 

The American College of Cardiology/American Heart Association (ACC/AHA) and European Society of Cardiology (ESC) have guidelines on the timing for operation of severe tricuspid regurgitation (TR), however, unlike MR there is not enough research on the appropriate timing for tricuspid valve (TV) repair. The guidelines are therefore not predicated on cumulative evidence [25, 26]. Many recent studies have suggested that early surgery is better since it improves short- and long-term post-operative outcomes [27, 28, 29]. A prior study suggested that restoring normal life expectancy did not occur even in patients with NYHA class II symptoms [30], emphasizing the need for TR surgical management before heart failure symptoms develop [31].

### 3.1 Etiology of Tricuspid Valve Regurgitation

The tricuspid annulus is comprised of a fibrous ring where the leaflets are attached. The tricuspid annulus area is 8~12 ${\mathrm{cm}}^{2}$ and is about 20% bigger than the mitral annulus.

**Fig. 4.**
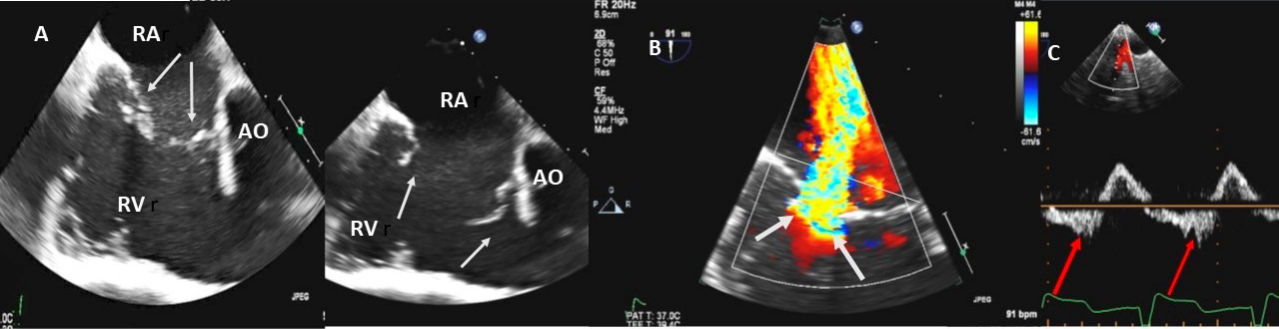
**Functional Tricuspid regurgitation**. (A) TEE mid esophageal inflow-outflow view shows a dilated RV and tricuspid annulus (white arrows). (B) TEE 4 chamber view color Doppler showing centrally directed severe tricuspid regurgitation with PISA (white arrows). (C) PW Doppler showing systolic hepatic flow reversal, which suggests severe TR (red arrows). RA; right atrium; RV, right ventricle; AO, aorta.

In adults TR is most commonly secondary (or functional), with normal leaflets and chords. Dilatation of the right atrium and RV with dilation of the tricuspid annulus is the most cause of secondary TR [32] (Fig. 4A–C). In an echocardiographic review of patients without primary tricuspid valve disease [33], severe TR was associated with higher pulmonary artery systolic pressure (PASP), atrial fibrillation, right atrial and RV enlargement, LV dysfunction, and primary mitral valve disease. In patients with concomitant mitral valve disease, TR is referred to as functional and is considered to be caused by filling pressure elevation in left heart, resulting in eventually tricuspid annular dilation and the tethering of tricuspid leaflets induced by RV enlargement [34, 35, 36]. This may also be true in patients with late TR after left side valve surgery [37]. Secondary TR in patients with right ventricular pressure and/or volume overload is caused by annular dilatation and increased tricuspid leaflet tethering [2].

Marked right atrial enlargement in atrial fibrillation with dilated tricuspid annulus and annular dynamics results in “atrial functional TR”. Moderate or severe TR was associated with most of these factors, as well as presence of an RV pacemaker lead, older age, and female sex. Primary pulmonary parenchymal diseases that elevate PASP and pulmonary arterio-venous disorders such as pulmonary embolism and primary pulmonary hypertension are other causes. Although TR severity was associated with higher PASP, many patients with pulmonary hypertension had only mild TR (65% of patients with PASP 50 to 69 mmHg and 46% with PASP ≥70 mmHg).

Primary tricuspid valve disease was found in 10 percent of patients with severe TR [38]. Iatrogenic or acquired TV diseases such as endocarditis, pacemaker/ICD leads, ruptured chords or flail leaflets in myxomatous tricuspid valve disease, chordal rupture from repeated right heart biopsies as in heart transplant recipients, and chest trauma are common causes of TR. It may result from mechanical causes that impair closure, such as scar formation or thrombus on the leads, although the perforation or laceration of the valve leaflets is another cause of TR. Lead impingement, lead adherence, and lead entanglement are TR causing pathophysiologies [39]. Another mechanism is asynchrony, which occurs with abnormal right ventricle activation caused by pacemaker [40, 41]. One study showed 5 of 41 (12%) of patients having tricuspid valve leaflet perforation or impingement by the PPM or ICD lead detected on the initial TTE [39]. Pacemaker or ICD leads causing damage to the tricuspid valve may result in severe symptomatic TR and may not be well visualized by TTE.

TR is often observed following orthotopic cardiac transplantation with a prevalence ranging from 67% to 85% in echocardiographic series [42]. Damage to the valve apparatus during endomyocardial biopsy could account for the high prevalence of TR in these patients [43]. TR may be caused directly by anatomic disruption of the valve apparatus, such as torn leaflet or ruptured chordae tendinea; and excessive leaflet motion is associated with severe prolapse or flail valve [44, 45]. With longer follow-up duration, the severity and clinical impact of TR worsens. TR can potentially exert hemodynamic stress on the right ventricle, which then undergoes morphologic remodeling that leads to chamber dilation. This may worsen right-sided atrioventricular coupling geometrically and hemodynamically that in turn causes more TR [45].

Marantic endocarditis in connective tissue disease, Ebstein anomaly, carcinoid syndrome, and drug-induced disease (combined use of the anorectic drugs, fenfluramine and phentermine, or the dopamine agonist pergolide) are other etiologies of primary TR.

**Fig. 5.**
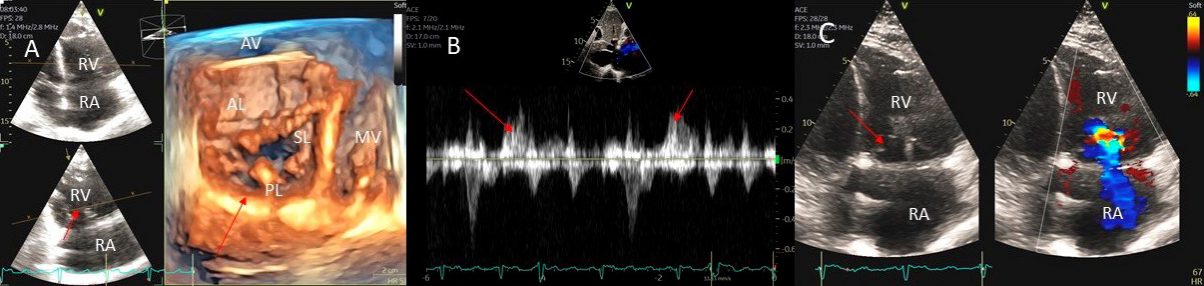
**Tricuspid regurgitation induced by pacemaker lead impingement**. (A) 3D transthoracic echo showing and dilated RV, RA and anterior leaflet impingement by the device lead (red arrows). (B) Inspiratory systolic hepatic flow reversal (red arrows), which suggests severe TR. (C) Apical four chamber view showed severe tricuspid regurgitation. RA, right atrium; RV, right ventricle; AV, aortic valve; AL, anterior leaflet; PL, posterior leaflet; SL, septal leaflet; MV, mitral valve.

**Fig. 6.**
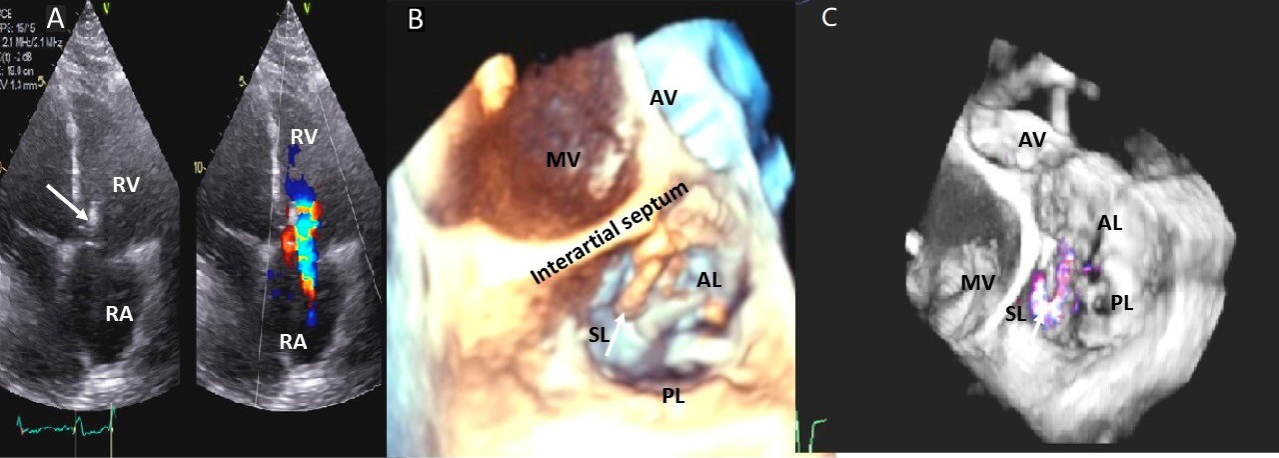
**Tricuspid regurgitation induced by pacemaker lead valve perforation**. (A) Transthoracic 2D 4 chamber view showing pacemaker lead going through the TV leaflet (white arrow) and causing TR. (B) 3D enface view of the TV from the right atrial perspective showing the pacemaker lead going through the margin of the septal leaflet (SL) of the TV (white arrow). (C) 3D color Doppler view of the TV from the atrial perspective showing origin of TR at the site of leaflet perforation. MV, mitral valve; AV, aortic valve; PL, posterior leaflet; AL, anterior leaflet

**Fig. 7.**
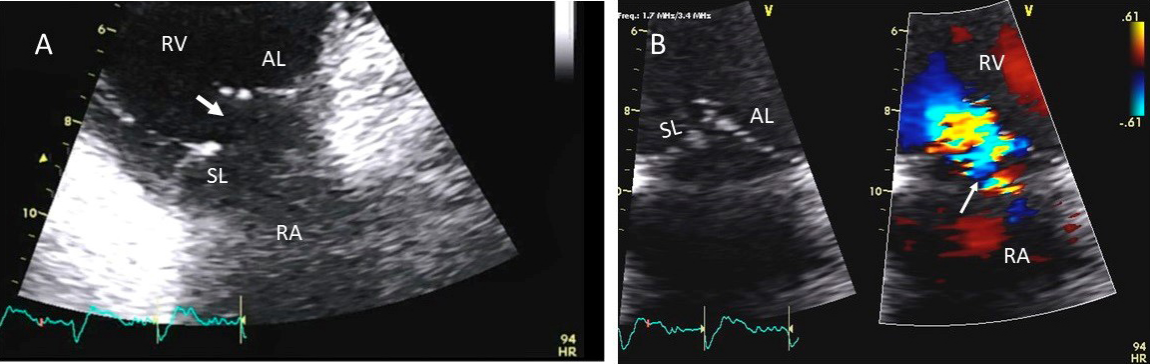
**Tricuspid regurgitation following endomyocardial biopsy**. (A) Flail tricuspid valve leaflet that occurred as a complication of an endomyocardial biopsy. Apical 4 chamber view showed flail septal leaflet (SL, septal leaflet; AL, anterior leaflet). (B) Color-flow Doppler imaging. Eccentric, anteriorly directed jet of tricuspid regurgitation (SL, septal leaflet of tricuspid valve; AL, anterior leaflet of tricuspid valve).

Examples of valvular injury directly from implantable cardioverter-defibrillator lead placement (Fig. 5A–C) or a permanent pacemaker (Fig. 6A–C) or endomyocardial biopsy in cardiac transplant recipients (Fig. 7A,B) are shown.

Congenital heart disease such as intracardiac shunts at atrial or ventricular level and anomalous pulmonary venous return cause RV volume and pressure overload causing RV enlargement and dilated tricuspid annulus. RV outflow obstruction such as in the pulmonic valve or pulmonary artery stenosis, tetralogy of Fallot and systemic right ventricle facing systemic afterload result in RV enlargement, hypertrophy, systolic dysfunction and increase in RV systolic pressure with resultant TR. Other causes include Epstein anomaly and primary RV dysfunction due to RV infarct and RV cardiomyopathies.

### 3.2 Echocardiography Evaluation of Tricuspid Valve

#### 3.2.1 Assessment of TR Severity

The annular diameter is measured and evaluated for tricuspid annular dilatation, and one study has shown TR development according to tricuspid annular circumference [46]. Unlike 2D echocardiography, 3D echocardiography is able to provide a clear definition of whole tricuspid annulus in about half of the patients [19] and has shown that the tricuspid annulus is bimodal in shape or “saddle shaped” similar to the mitral annulus. There are 4 superior points on the right atrial side and the anterior and posterior aspects of the annulus and 2 inferior points in the right ventricle at the medial and lateral aspects of the annulus [47, 48, 49]. As functional tricuspid regurgitation develops, the tricuspid annulus gets more planar and circular, and expands anterolaterally [47, 48].

The TR severity was assessed using the maximum TR jet area and right atrium size in the apical four chamber and right ventricular inflow views. TR area to right atrial area ratio was placed into four groups, (1) the ratio <10% considered as grade 1+, (2) the ratio 10–20% as grade 2+, (3) the ratio 20–40% as grade 3+, (4) and the ratio >40% as grade 4+ [7, 46]. ASE recommends >50% ratio for severe TR. Severity of TR is determined by integration of several imaging and Doppler findings. The major Doppler criteria for severe TR are TR EROA of ≥0.4 ${\mathrm{cm}}^{2}$, regurgitant volume of ≥45 mL, a vena contracta width of ≥0.7 cm and hepatic vein systolic flow reversal [5]. However, hepatic vein systolic flow reversal may be misleading unless sinus rhythm is present, as normal atrial filling during ventricular systole depends on a preceding normal atrial contraction [5]. Marked diastolic flattening of interventricular septum due to increased volume within the RV (diastolic overload) is often seen in severe TR whereas systolic and diastolic flattening of the septum is seen in patients with pulmonary hypertension with secondary TR. 3D color Doppler assessment of TR demonstrated usefulness in measuring the vena contracta of the tricuspid regurgitant jet by 3D color Doppler data [50]. TR jet envelope echo Doppler density, depicts volume of blood flow and hence the signal may be as dense as forward CW tricuspid inflow in severe TR. Large tricuspid leaflet coaptation gap is often seen in patients with severe TR with marked annular dilation. Early peaking and triangular dense TR envelope also indicate severe TR although triangular TR envelope can be seen in the absence of severe TR due to elevated right atrial pressure. In severe TR ventricularization of atrial pressure occurs with low TR jet peak velocity [6]. Similar to assessment of MR, no one parameter should be relied on entirely for TR assessment and assessment of severity should be based on multiple echo parameters.

#### 3.2.2 Prevention of TR Pathogenesis and Treatment of TR in Patients Undergoing Mitral Valve Surgery

In patients with concomitant mitral valve disease undergoing surgery, tricuspid annuloplasty with or without an annular ring has been widely performed with the intention of reducing tricuspid annular diameter and ultimately of eliminating significant TR [51].

According to current evidence, tricuspid annular dilatation was considered an indication for prophylactic tricuspid repair, regardless of the severity of TR. The definition of tricuspid annular dilatation during surgery does not have enough data, and there is only one study which reported a threshold of 83 ${\mathrm{mm/m}}^{2}$ intraoperatively with good clinical result [46].

When undergoing left-sided valve surgery even in patients with mild or no TR, intraoperative measurement of the tricuspid annular circumference as well as annular diameter indexed for body surface area predicted TR progression [52, 53]. European guidelines recommend tricuspid valve surgery in moderate primary TR or in patients with mild to moderate secondary TR when the annular dimension is >40 mms (21 ${\mathrm{mm/m}}^{2}$) in patients who are undergoing left sided valve surgery [50].

To reduce the incidence of cardiac pacemaker lead placement related TR, TEE-guidance was found safe and feasible during PM or ICD implantation and resulted in steps to optimize lead position. At discharge, lead position was stable, and TEE-guided implantation was associated with less worsening of TR than standard lead implantation guided by fluoroscopy [54]. In this study, leads were placed according to a dedicated echo protocol with focus on a trans gastric *en face* view of the tricuspid valve targeting a stable lead position in a tricuspid valve commissure (preferentially postero-septal) and an apical ventricular lead position [54]. Echocardiography, particularly 3D TEE) is helpful in identifying and defining the mechanism of pacer-lead-related TR [55] (Fig. 6).

#### 3.2.3 TR Post Orthotopic Cardiac Transplantation

Echocardiography permits the direct visualization of the endocardial border and identification of the ventricular septum, apex, and free wall, as well as the tricuspid valve and subvalvular apparatus during biopsy procedure. This visualization allows for the early detection of complications such as a pericardial effusion or damage to the tricuspid valve or subvalvular apparatus as well as assessment of cardiac function [56]. One study in 183 patients who had undergone a total of 2,960 biopsies for an average of 16.2 biopsies per patient showed that over a mean follow-up period of 4.22 years, severe TR accompanied by flail components of the tricuspid valve was found in as many as 7% of patients [57]. The *en face *view of the TV obtained by RT-3DE allows visualization of chordal rupture and of the concomitant annular dilation [58]. Recipients with significant TR are more symptomatic and have poorer right-sided heart function and renal function compared with those with mild or no TR post cardiac transplant [59].

## 4. Aortic Valve 

Unlike mitral and tricuspid valves, the semilunar aortic valve supporting structure is less complex but similar to atrioventricular valves, aortic valve disease can be primary involving aortic leaflet pathology or secondary due to the dilation of the aortic sinuses or the ascending aorta or ventriculo-arterial connection. Aortic leaflet pathology is a more common cause of significant aortic regurgitation (AR).

### 4.1 Common Etiologies of Aortic Valve Regurgitation

I Dilation of aortic root/ascending aorta: (a) sinotubular junction and ascending aorta, (b) sinuses and ST junction, (c) ventriculo-arterial dilation at the aortic annulus resulting in aortic leaflet malcoaptation.

**Fig. 8.**
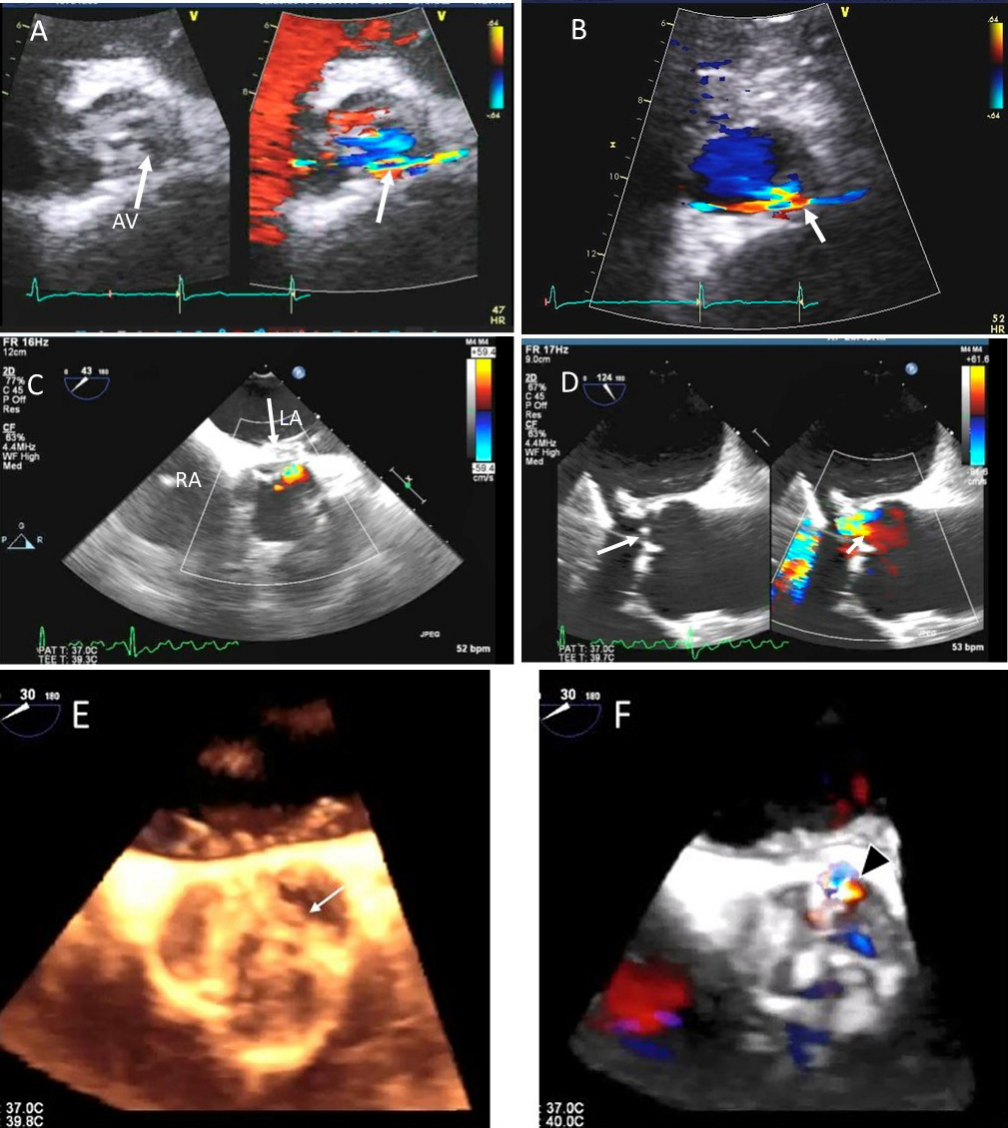
**Aortic regurgitation and left coronary cusp perforation**. (A) TTE color Doppler parasternal short axis view demonstrating left coronary cusp perforation (white arrows), resulting in aortic regurgitation. (B) TTE color Doppler short axis view showing origin of aortic regurgitation through the left coronary cusp perforation (white arrow). (C) TEE color Doppler short axis view showing a better delineation of the origin of aortic regurgitation jet through the left coronary cusp perforation (white arrow). (D) TEE long axis view demonstrating the perforated coronary cusp on 2D (white arrow) with aortic regurgitation jet originating through the perforation (white arrow) and not through the aortic leaflet coaptation. (E) 3-dimensional (3D) TEE short axis view of the aortic valve showed a clear definition of the left coronary cusp perforation (white arrow). (F) 3-D TEE color Doppler short axis view showing aortic regurgitation jet originating through the left coronary cusp (black arrowhead). Direct planimetry of color Doppler aortic regurgitant orifice can be performed online or via offline post processing of 3D data sets without geometrical assumptions of PISA method or continuity equation.

**Fig. 9.**
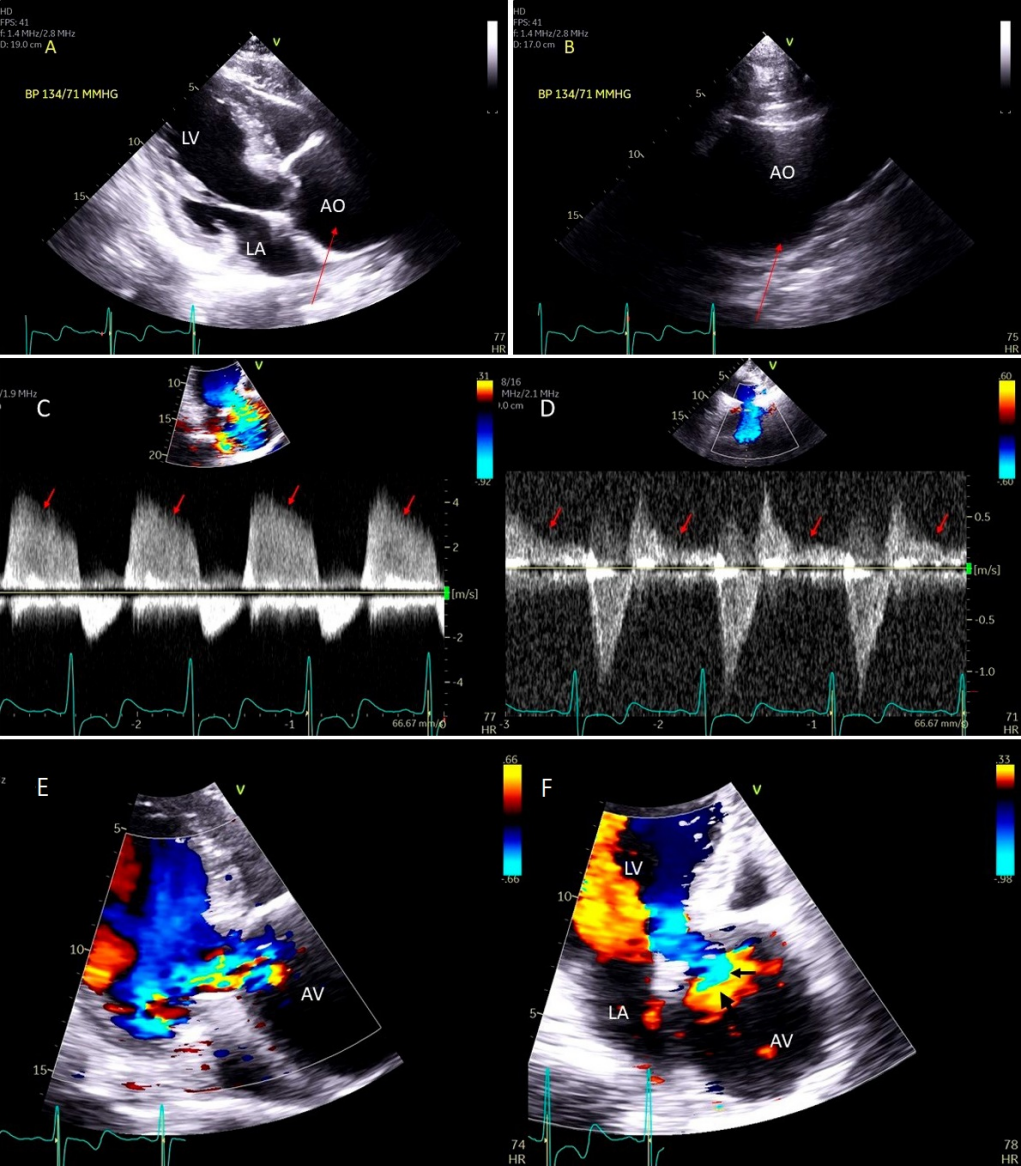
**Aortic valve regurgitation induced by dilated annulus**. (A) Transthoracic echocardiogram parasternal long axis view showing markedly dilated aortic sinuses (red arrow). (B) Imaging at a higher parasternal window shows aneurysmal aortic sinuses (red arrow) and normal ascending aorta above sinotubular junction. (C) Three chamber view with continuous-wave (CW) Doppler showing dense AI Doppler envelope with a steep deceleration slope (red arrows) suggesting severe AI. (D) CW Doppler recording at the proximal descending thoracic aorta demonstrating pandiastolic flow reversal (red arrows), another feature of severe AI. (E) Color Doppler parasternal long axis view showing AI color jet occupying two third of the LV outflow tract with a wide vena contracta at origin from the aortic valve leaflets (>0.6cm) – two other features of severe AI. (F) Color Doppler apical 3 chamber view showing measurement of AI flow convergence radius (black arrows) to calculate effective regurgitant orifice area by proximal isovolumic flow acceleration method.

II Aortic cusp prolapse/perforation: endocarditis, aortic dissection (Figs. 8, 9)

III Restrictive Valve Disease:

a. Degenerative—Calcification of aortic leaflets with aging

b. Congenital—Bicuspid aortic valve

c. Rheumatic

### 4.2 Echocardiographic Assessment of Aortic Valve and Regurgitation

TTE is an essential first diagnostic tool and is frequently sufficient to assess the presence and severity of AR [60]. Besides an assessment of the aortic valve and aortic root anatomy to determine the etiology of the regurgitation, assessment of AR should include evaluation of LV size, geometry, and function [5]. In chronic AR, TTE is essential in monitoring the changes in LV geometry (LV size and LV volume increase) and function (progressive worsening LV function) due to the prolonged LV volume overload. LV dilatation, particularly with preserved LV function, is a supportive sign of significant AR and becomes more specific excluding other causes of LV volume overload (e.g., in athletes, anemia). In severe acute AR, the LV is not dilated, and the LV end-diastolic pressure increase may cause the MV premature closure, best documented with an M-mode.

Ratio of AR jet width to the LVOT width of ≥65% (Fig. 9), vena contracta of AR jet (≥0.6 cm), effective regurgitant orifice area of ≥0.3 ${\mathrm{cm}}^{2}$ (Fig. 8), regurgitant volume of ≥60 mL by PISA method [or continuity equation], density of AR jet Doppler velocity similar to aortic valve forward flow, pressure half time of AR jet <200 ms (Fig. 9C), diastolic flow reversal in the abdominal aorta and pan-diastolic flow reversal in the proximal thoracic aorta with time velocity integral of >15 cm (Fig. 9D), are all used as criterial for severe AR. Importantly no one criterion is diagnostic and an integrative approach using multiple methods should be used to arrive at a conclusion on AR severity. LV enlargement should be present in patients with chronic severe AR.

TEE is usually indicated for assessment of mechanism of AR, exclusion of aortic valve infective endocarditis, measurement of aortic root size and quantitation of AR severity when not feasible by TTE. Both 2D and 3D TEE have shown utility, improving diagnostic accuracy of TTE in AR related condition: infectious or inflammatory endocarditis, isolated aortic root dilatation, or acute aortic dissection [61].

The diagnostic value of 2D and 3D TEE in defining the mechanisms of AR is particularly important for pre-operative evaluation of patients undergoing aortic root surgery, or valve repair or replacement [62].

**Fig. 10.**
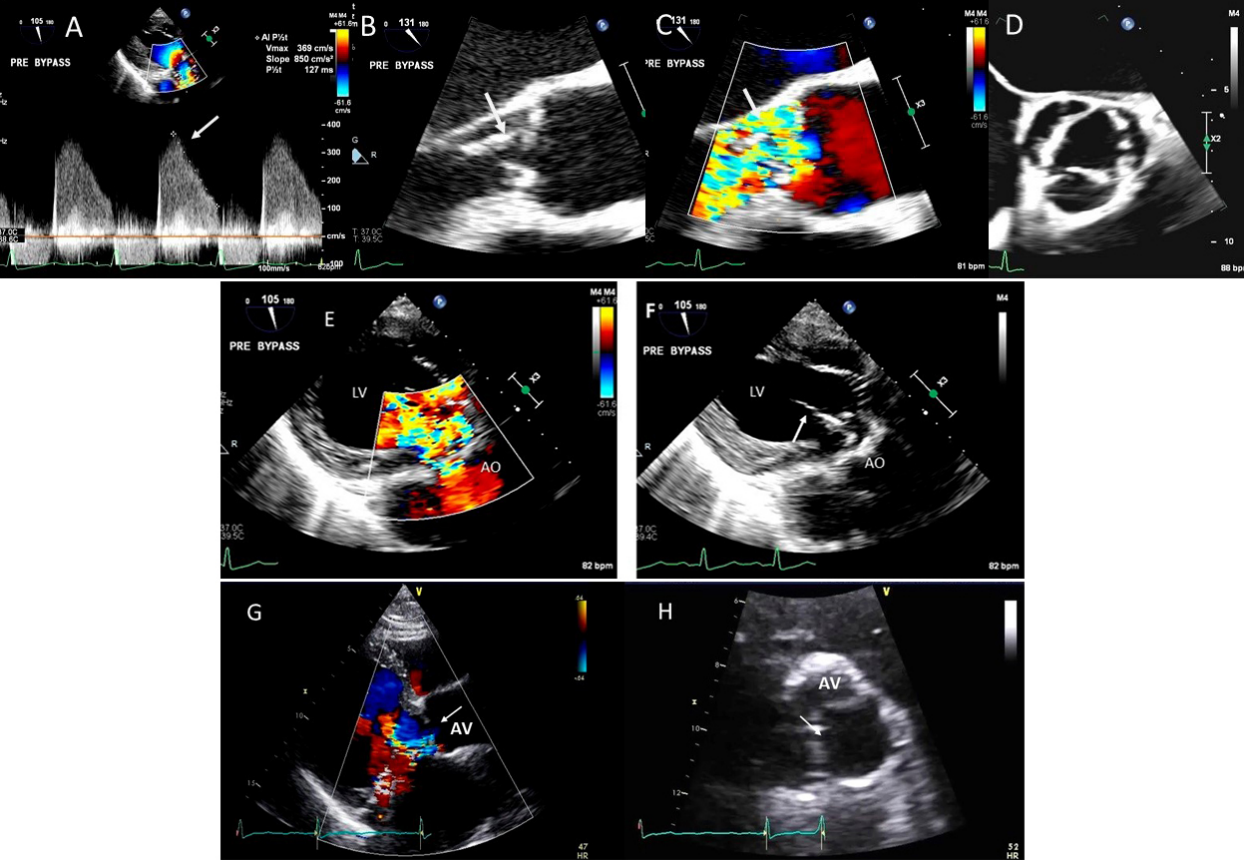
**Endocarditis induced aortic cusp tear and aortic regurgitation (AR) demonstrated by TEE:** (A) TEE transgastric (TG) view showing dense CW Doppler signal of AR with a steep slope, (B) Mid esophagel (ME) long axis view showing torn and flail aortic valve cusp, (C), same view showing severe AR on color Doppler, (D) ME short axis view showing a tricuspid aortic valve, (E) TEE TG view showing dilated LV and severe AR on color Doppler and (F) torn AV leaflet on 2D. (G) transthoracic parasternal long axis view showing a dilated LV and poorly defined AR on color Doppler, (H) transthoracic AV short axis view with an ill-defined view showing possible of the torn left coronary cusp (white arrow).

Common causes of leaflet malfunction causing AR include degenerative leaflet calcifications, and infective endocarditis (Fig. 10), bicuspid aortic valve perforation and rheumatic fever. The causes of AR include Marfan’s syndrome, annulo-aortic ectasia (idiopathic root dilatation) (Fig. 9), aortic dissection, connective tissue disease, and syphilis. The Carpentier classification is also widely used to describe the mechanism of AR [63].

Using 2D biplane, 3D and 3D color Doppler, the exact perpendicular plane to the aortic regurgitation jet can be identified, from which planimetry of the AV coaptation gap as well of the color Doppler vena contracta can be performed [64]. This has been shown to have a good correlation with aortographic grading of AR. When the shape of the regurgitant orifice is nonsymmetric, by using 3D images, invalid geometric assumptions of the vena contracta can be avoided with direct measurement [65]. 3D echocardiographic color Doppler also allows visualization, and measurement of multiple jets and correlated morphologically with surgical findings [65].

Isolated aortic valve perforation can occur post endocarditis or post cardiac surgery [15, 16, 17]. Restriction of aortic valve leaflet motion may occur due to leaflet tethering [18, 19]. Combined use of 2D, color Doppler and 3D TEE may facilitate location and mechanism of AR and can allow valve repair instead of replacement.

The time duration of Doppler flow of subclavian artery into late diastole is reported to be another important parameter for the severity of AR [66].

Aortic stenosis (AS) is commonly combined with AR (in nearly 80% of cases) but the regurgitation is usually only mild or moderate in severity and measures of AS severity are not significantly affected [5]. When severe AR combines with AS, measuring of AS severity remain accurate including maximum velocity, mean gradient, and valve area. However, because of the high transaortic volume flow rate, maximum velocity, and mean gradient are higher than expected for a given valve area [5].

## 5. Pulmonic Valve Regurgitation

### 5.1 Etiology of Pulmonic Valve Regurgitation

Significant pulmonary valve regurgitation (PR) in adults is generally seen in those with prior history of pulmonic valve surgery for congenital heart disease. PR can also result from significant pulmonary hypertension of any cause.

### 5.2 Echocardiographic Assessment of PR 

**Fig. 11.**
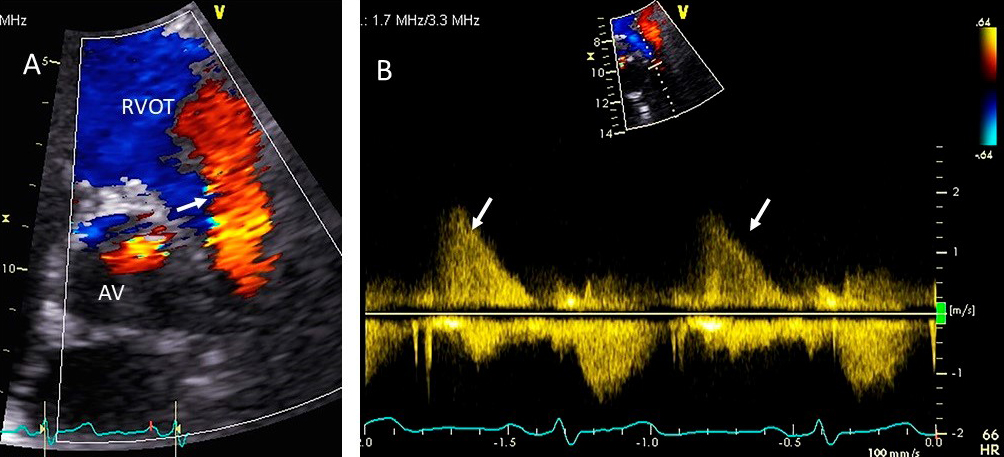
**Pulmonic valve regurgitation**. (A) TTE basal short axis view showing color Doppler of pulmonic valve (in the long axis view) and aortic valve in the short axis view. There is severe pulmonic regurgitation as shown by a wide diastolic jet in the RVOT occupying >60% of RVOT (white arrow). This jet was very short only visible in 2 frames. AV, aortic valve; RVOT, right ventricular outflow tract. (B) Corresponding CW Doppler showing dense PR signal with rapid deceleration of PR velocity to baseline (white arrows) so that the PR signal terminates before the onset of the next cardiac cycle.

PR is diagnosed with color Doppler revealing diastolic flow in the RV outflow tract (Fig. 11). In severe PR with normal PA pressures (e.g., primary PR), the color jet has low velocity with laminar flow and the duration of the flow can be misleading, as severe PR may lead to rapid equalization of the RV pressure with diastolic pulmonary artery pressure, and thereby lead to a very short duration of flow [67]. In secondary PR from pulmonary hypertension, the jet is aliased and usually holodiastolic [68]. Severe PR from primary or secondary causes has an intense spectral Doppler signal. Other parameters to assess PR severity include PR jet width: if it occupies >50–65% of the RVOT, this suggests severe PR, whereas a narrow jet <25% of the pulmonary annulus suggests mild PR. This may be less reliable in the setting of an eccentric jet which may underestimate the severity of the regurgitation. Another helpful parameter is the pressure half time (PHT). A short PHT, defined as <100 ms)is consistent with severe PR. Early termination of the PR flow would be seen with varying degrees of late diastolic forward flow in the pulmonary artery due to increase in RV pressure relative to the pulmonary artery pressure [69]. However, a short PHT can also be seen in the presence of elevated diastolic intrapulmonary pressures as well as RV diastoldys function [70, 71]. Other quantitative variables such as regurgitant volume and fraction can also be used however the difficulty in measuring the pulmonary annulus size makes these measurements less accurate. Additionally, they have not been validated as they have been with AR.

**Fig. 12.**
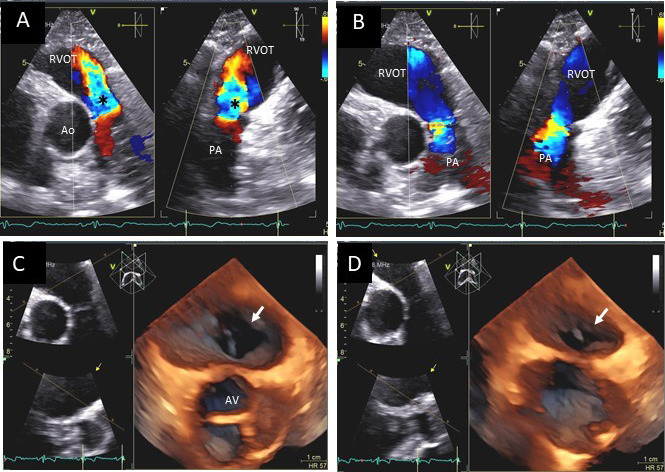
**Pulmonic valve regurgitation and stenosis**. Transthoracic echocardiographic images in a patient with carcinoid syndrome involving the pulmonic valve. Short axis biplane color Doppler views at the cardiac base showing severe pulmonic insufficiency (black asterisks - (A) and turbulent forward flow in systole due to pulmonic stenosis in (B). Three dimensional transthoracic views showing diffusely thickened and restricted pulmonic valve leaflets in systole (white arrow - (C)) and incomplete closure of the thickened pulmonic valve leaflets in diastole (white arrow - (D)) causing severe pulmonic regurgitation.

Severe PR also causes RV dilatation and diastolic flattening of the interventricular septum due to volume overload. A normal RV size suggests the absence of chronic severe PR. Carcinoid disease causes thickening and restriction of tricuspid and pulmonic valves leading to valve stenosis and regurgitation. This leads to turbulent forward flow in systole due to varying degrees of pulmonic stenosis and regurgitation due to incomplete closure of the thickened and restricted valve leaflets (Fig. 12).

## 6. Conclusions

Echo plays a vital role in assessing severity and mechanism of cardiac valve regurgitation. It is important for the echocardiographer to report not only valve regurgitation severity by integrative approach using qualitative, semiquantitative as well as quantitative methods on TTE but also comment on the potential mechanism of valve regurgitation when evident. The hemodynamic effects of regurgitation on cardiac geometry and function should be reported, in light of the ACC/AHA/European guideline recommendations. There should also be a comment on its whether TEE will improve assessment of valve regurgitation severity or mechanism. A comment on additional testing such as exercise echo, dobutamine echo, next echo follow up and other non-echo imaging testing recommendations may be considered to guide the physician on subsequent management.

## 7. Summary 

Three-dimensional (3D) echocardiography has become one of the most promising methods for the diagnosis of valvular heart disease, and recently has become an integral clinical tool since the development of high quality real-time transesophageal echocardiography (TEE). 3D echocardiography provides incremental information over standard 2D techniques. In particular, 3D echocardiography has proven to be the most feasible and reliable method for understanding the complex mitral valve anatomy and etiology of mitral regurgitation. 3D TEE has been useful for surgical management of mitral valve disease in particular for mitral valve repair. 3D TEE is pivotal for nonsurgical mitral procedures such as edge to edge mitral repair and transcatheter closure of paravaluvular leaks. In addition, color Doppler 3D echo has been valuable to identify the location of the regurgitant orifice and the severity of the mitral regurgitation. 3D acquisition on top of a standard 2D study typically requires only a few extra minutes of imaging. Live 3D imaging has become more useful and may even obviate the need, in some situations, for post-processing of 3D data sets that requires some additional time. For aortic and tricuspid valve diseases the usefulness of 3D echo is recognized for certain situations. 3D can help better define the etiology of aortic valve regurgitation such as cusp perforation or tethering/restriction of aortic valve leaflets or paravalvular regurgitation. Aortic annulus area can be measured more precisely, allowing 3D annular reconstruction for selecting correct aortic valve size during percutaneous aortic valve implant procedures. Regarding tricuspid valve, 3D provides *en face* views of the tricuspid valve allow determining if the tricuspid regurgitant jet origin is a coaptation leak due to tricuspid valve malcoaptation or is due to pacemaker and/or ICD leads impinging upon or perforating one of the tricuspid leaflets. 3D also allows determination of location of pacemaker/ICD lead vegetations and if the tricuspid valve apparatus is involved. Tricuspid valve 3D anatomy can be adequately assessed by transthoracic technique and may not require a TEE. 3D TEE will be an integral part of the evolving percutaneous tricuspid valve repair/replacement procedures. Continued development of 3D TEE ultrasound technology, will continue to simplify and enhance this technology and increase its adoption, making its use more widespread to refine the diagnosis and better guide the treatment of patients with valvular heart disease.
